# Suicide Attempt as the Presenting Symptom in a Patient with COVID-19: A Case Report from the United States

**DOI:** 10.1155/2020/8897454

**Published:** 2020-09-16

**Authors:** Nelson Telles-Garcia, Tyler Zahrli, Gaurav Aggarwal, Sakshi Bansal, Leonard Richards, Saurabh Aggarwal

**Affiliations:** ^1^Department of Medicine, UnityPoint Health, Des Moines, IA, USA; ^2^Broadlawns UnityPoint Psychiatry Residency, Des Moines, IA, USA; ^3^Department of Medicine, Jersey City Medical Center, Jersey City, NJ, USA

## Abstract

Coronavirus disease 2019 (COVID-19) has attained a pandemic status and is associated with a high morbidity and mortality. Social isolation, fear of ostracization, and illness itself and limited access to care can lead to worsening of mental illnesses. We report a case from the United States describing a young male with a suicidal attempt who was subsequently found to have COVID-19 infection. Further research is needed to evaluate potential factors for this unique association.

## 1. Introduction

The novel coronavirus disease 2019 (COVID-19), caused by severe acute respiratory syndrome-coronavirus 2 (SARS-CoV2), that was first reported in China has quickly spread globally achieving a pandemic status, accounting for more than 1.5 million cases and close to 100,000 deaths worldwide [1, 2]. Viral illnesses are known to be associated with significant physical morbidity and mortality but also have significant impact on mental health. Coronavirus and influenza seropositivity has been previously shown to be associated with psychiatric findings including suicidality [3]. However, the impact of COVID-19 infection on suicidal tendency has not been reported. We report a case of a patient with suicidality in a patient infected with COVID-19.

## 2. Case Report

A 38-year-old American man with past medical history significant for hypertension, diabetes mellitus, obesity, and cervical spine disk bulge and a past psychiatric history significant for anxiety and depression self-presented to our hospital's emergency department (ED) after a suicide attempt. He ingested 10-15 tablets of baclofen 10 mg each prior to arrival. The patient reported suicidal ideation with intent after suicidal behavior with possible injuries. He recently moved to Iowa from Minnesota, started a new job and found a new apartment over the past two weeks. Apart from worsening depression and active suicidal ideation with intent, he complained of recent worsening of headaches, neck pain, “feeling warm,” and mild nausea. Patient denied prior history of any suicide attempts, homicidal ideation, psychosis, or manic symptoms. The patient had seen a psychiatrist two months prior and denied depressive symptoms at that time. He reported three prior psychiatric hospitalizations for worsening mood symptoms, once requiring electroconvulsive therapy (ECT). The patient reported medication adherence that providers confirmed with pharmacy. The patient recently moved in with his cousin in Iowa and was single. He reported good family support and financial resources. Liabilities included recent move, physical illness complaints, living situation, and chronic mental illness. Of note, the patient reported two paternal family members who completed suicide as well as maternal and paternal family history of depression.

In the emergency department, the patient's vitals were temperature 99.5°F (37.5°C), blood pressure 107/60, respiratory rate 18 breaths/min, and oxygen saturation 97%. He was tachycardic with HR 123 beats per minute. The patient's physical exam was normal both in the emergency department and during the initial history and physical except tachycardia. Significant laboratory data in the ED showed thrombocytopenia with platelets 11 × 10^3^/*μ*L (Ref: 150–450 × 10^3^/*μ*L mild hyponatremia, sodium 131 mmol/L (Ref: 126–135 mmol/L) elevated alanine transaminase level 55 U/L (Ref: <41 U/L), and normal aspartate transaminase level. Blood alcohol level was negative. Urine drug screen was positive for methadone. The patient was admitted under psychiatry service and diagnosed with severe, recurrent major depressive disorder.

A few hours after admission, the patient was noted to be febrile with temperature measured 101.3°F (38.5°C). On repeat questioning, he admitted to a mild dry cough over the last 12 hours. He denied any recent travel history or exposure to sick contacts. Medicine service was consulted, and the patient was transferred to the general medical floor. Given his symptoms, a nasopharyngeal swab was sent as part of COVID-19 surveillance, which came back positive for SARS-CoV-2 on polymerase chain reaction (PCR) assay. The patient was put in an isolation room, and proper precautions were taken to prevent spread to healthcare workers. However, the situation was challenging due to inability to perform comforting measures like hand holding and engage in conversations from close distance. The patient was discharged on day 5 for self-quarantine at home.

## 3. Discussion

This case describes a patient with known anxiety and depression who was admitted for a suicide attempt and was diagnosed with COVID-19 infection during the same hospitalization. The patient had no prior history of suicide attempt prior to this encounter. To the best of our knowledge, this is among the first reports of suicidal ideation in a patient with COVID-19 infection from the United States.

COVID-19 has been demonstrated to be associated with high morbidity and mortality. The three most common symptoms reported by patients infected with COVID-19 are fever, cough, and dyspnea. Less common symptoms are myalgia, anorexia, malaise, sore throat, nasal congestion, and headache. Symptoms may appear in as few as two days or can take as long as 14 days after exposure. Anecdotally, mental disorders have been linked to infection with common respiratory viruses [4]. However, only influenza B has been reported to be associated with suicide attempt in patients with coexisting mood disorders [3].

Coronaviruses are negatively stranded RNA viruses, with the capacity of rapid mutation and recombination. Generally, two proposed pathophysiologic mechanisms explain the bidirectional relationship between viral illness and psychiatric illness. First, the virus can be directly toxic to the brain. They are capable of replication within the nervous system; the presence of viral RNA has been previously demonstrated in the brain tissue of patients with multiple sclerosis [5, 6]. Common respiratory viruses such coronaviruses and influenza viruses are neurotropic and have also been isolated from the central nervous system [7].

Secondly, the immune response generated by the host in response to viral illness can also affect mood disorders through indirect neurological effects [8]. Most of the symptoms are thought to be related to the effect of specific cytokines, including tumor necrosis factor alpha, interleukin 6 and 8, and interferon alpha. These immune responses ultimately result in a systemic inflammatory process like the physiological consequences of stress and mood disorders. The immune response to coronavirus involves cell-mediated and humoral-mediated immunities, with production of antibodies, interferon, and cytokines. Increased level of antibodies to prior existing strains of coronavirus has been described in a case report in a patient with recent onset of mental illness [9].

Symptoms such fatigue, lack of appetite, decrease of social interaction, and loss of interest can be seen in both scenarios [10]. However, the common symptoms of depression such as hopelessness, worthlessness, guilt, and suicidal ideation are typically not seen in infectious processes alone. High stress, negative affect, and depression reduce cell-mediated immunity, ultimately predisposing to other illnesses such as viral illness. This creates concern of self-perpetuating illness during the current COVID-19 pandemic.

Recent Chinese publications have expressed concerns regarding clusters of COVID-19 breakouts among those with mental illness [11]. China has also found increased rates of negative emotions such as anxiety and depression and decreased positive emotion during the country's epidemic [12]. Social isolation, stress over the pandemic, and negative emotions make individuals more susceptible to COVID-19 infection by enhancing a stress-related immunocompromised state, which can further contribute to worsening mood symptoms. Financial burden, unemployment, and domestic abuse can be some of the reasons related to increase in suicidal tendencies with COVID-19 [13]. Also, the infodemic of fake news and misinformation can make people fearful of symptoms and increase stress. Our patient had multiple life stressors prior to worsening depressive symptoms and suicide attempt as well as prior to emergence of COVID-19 symptoms. In the first reported case in literature, a male from Bangladesh was deemed to have committed suicide due to concerns that he had COVID-19 infection and social ostracization [14]. Since then, other reports have surfaced from Asia and Europe demonstrating suicidal tendency in patients with COVID-19 infection [15–18]. Women and racial minority groups have been shown to be at higher risk for such behavior. The only other report from United States has been from New York where command hallucinations were found to be the first manifestation of COVID-19 [19].

With the ongoing COVID-19 pandemic, health care providers must recognize the relationship between mental illness and viral illness to better treat patients. Although this case cannot support causation, it does stress the bidirectional effects that physical and mental illness share. Certainly, this patient had social stressors prior to his suicide attempt; however, coexisting COVID-19 infection could have exacerbated the effects of these social stressors on his mental health and vice versa. Health care professionals and society should take note of these associations with the current pandemic and its ramifications.

## 4. Conclusion

We present a case of 38-year-old male who presented with his first suicidal attempt and was subsequently diagnosed to have COVID-19 infection. Factors underlying this association need to be further evaluated.

## Data Availability

Not applicable.
